# TIMP3 overexpression in myeloid lineage alleviates pancreatic damage and confers resistance to the development of type 1 diabetes in the MLDS -induced model

**DOI:** 10.3389/fendo.2023.1297847

**Published:** 2024-01-19

**Authors:** Viviana Casagrande, Stefano Menini, Chiara Internò, Giuseppe Pugliese, Massimo Federici, Rossella Menghini

**Affiliations:** ^1^ Department of Systems Medicine, University of Rome Tor Vergata, Rome, Italy; ^2^ Department of Clinical and Molecular Medicine, Sapienza University, Rome, Italy; ^3^ Center for Atherosclerosis, Department of Medical Sciences, University Hospital Policlinico Tor Vergata, Rome, Italy

**Keywords:** pancreas, diabetes, TIMP3, myeloid cells, insulitis

## Abstract

**Introduction:**

Type 1 diabetes mellitus (T1DM) development involves a complex interplay of genetic, environmental, and immunological factors. By modulating the activity of proteases and receptors, the protein tissue inhibitor of metalloproteinase 3 (TIMP3) plays a role in limiting the expression and function of pro-inflammatory cytokines, which have been implicated in the advancement of T1DM. This study was aimed at examining the effect of TIMP3 overexpression in myeloid cells on the development of T1DM.

**Methods and results:**

Twelve weeks after multiple low doses of streptozotocin (MLDS) treatment, diabetic mice overexpressing TIMP3 specifically in myeloid cells under the CD68 promoter (MacT3 mice) showed improved insulin secretion, islet morphology and vascularization, antioxidant defense system, and regulatory factors of mitochondrial biosynthesis and function. To get mechanistic insights into the origin of this protection, the severity of insulitis and inflammatory parameters were evaluated in pancreatic tissues 11 days after MLSD treatment, showing significantly reduced insulitis and levels of the pro-inflammatory cytokine tumor necrosis factor-α, interleukin -1β, and interferon -γ in MacT3 mice.

**Discussion:**

The results indicate that TIMP3 is involved in maintaining islet architecture and functions, at least in part, through modulation of pro-inflammatory cytokine production associated with insulitis and may represent a novel therapeutic strategy for T1DM.

## Introduction

Type 1 diabetes mellitus (T1DM) is an autoimmune-driven condition characterized by the targeted depletion of pancreatic *β*-cells, which are responsible for insulin production. It constitutes 5–10% of the total diabetes cases (1). Development of novel strategies against T1DM is an urgent need due to the limited therapeutic approaches already available. The insufficient production of insulin, resulting from damage to the cells responsible for insulin synthesis in the pancreatic islets, is a critical factor in the onset of DM (2). Various pathophysiological mechanisms, including enhanced inflammation, oxidative stress, apoptosis and necrosis have been involved in the pathogenesis of T1DM. The pathological state begins with an inflammation of the islets of Langerhans, known as insulitis, a crucial aspect of T1DM. This process leads to lymphocytic infiltration into the pancreatic islets, ultimately resulting in the autoimmune deterioration of *β*-cells (3). Myeloid cells, including macrophages residing in the pancreas and monocytes recruited from the peripheral blood during insulitis affect islet microenvironment and β cell function (4, 5) and may represent a potential therapeutic tool to regulate myeloid-derived cytokines production. Tissue Inhibitor of Metalloproteinase 3 (TIMP3) is a protein that binds to the Extracellular Matrix (ECM). It has the ability to inhibit the activity of membrane-bound MMPs, transmembrane MMPs, and sheddases. TIMP3 plays a crucial role in various physiological processes, including inflammation, fibrosis, and apoptosis (6). We previously demonstrated the role of TIMP3 in regulating glucose homeostasis and managing inflammation in metabolic tissues. This involvement has been observed in both genetic and nutritional mouse models of obesity and atherosclerosis, as well as in individuals with obesity-associated type 2 diabetes mellitus (T2DM) and atherosclerosis (7, 8).. Previously, we established a mouse model featuring CD68-dependent overexpression of TIMP3, referred to as MacT3. Our research revealed that MacT3 mice display resistance to inflammation induced by obesity, along with related metabolic disorders (9). Notably, this protective impact also encompasses atherosclerosis (10) and the onset/progression of diabetic kidney disease (11). These findings suggest that augmenting TIMP3 levels through myeloid lineage overexpression could present a promising therapeutic strategy for tackling meta-inflammation associated with cardiometabolic diseases and their complications. In our previous investigations, we consistently observed a significant finding: MacT3 mice demonstrated nearly half the susceptibility to hyperglycemia following a multiple low-dose streptozotocin (MLDS) treatment (45% of MacT3 mice exhibited hyperglycemia compared to 87% of wt mice) (11). This observation suggests that the enhanced expression of TIMP3 in myeloid lineage cells could potentially confer a protective effect in the endocrine pancreas (12). However, the mechanism was not investigated. This study aimed to evaluate the anti-diabetic effect of TIMP3 overexpression in MLDS induced model of T1DM.

## Materials and methods

### Animals and ethics statement

Animal studies were approved by the University of Tor Vergata Animal Care and Use Committee and Ministry of Health, license no. 378/2016-PR and 36/2019-PR. MacT3 transgenic mouse model was previously described and littermate C57BL/6J mice (wt) were used as controls (9, 10). Genotyping of the animals was analyzed using polymerase chain reaction (PCR) and diabetes was induced by a multiple low-dose streptozotocin (STZ) (Merck) injection (**Supplementary Materials and Methods**).

### Protein analysis

Pancreas were homogenized in 1% Triton X-100 ice cold buffer and 40 μg of total protein was subjected to electrophoresis (**Supplementary Materials and Methods**).

### RNA isolation and gene expression analysis

Total RNA was isolated from pancreas with Trizol reagent (Invitrogen Corp), reversed transcribed into cDNA and real-time PCR was performed (**Supplementary Materials and Methods**).

### Histopathological, immunohistochemical and immunofluorescence analysis

The pancreas was harvested, fixed in phosphate-buffered paraformaldehyde solution [4% (vol./vol.), halved, and then embedded in paraffin for histological and immunohistochemical (IHC) analysis, or in cold Tissue-Tek OCT Compound for immunofluorescence (IF) analysis (**Supplementary Materials and Methods**).

### Insulitis score

To semi-quantify the severity of insulitis at least 20 sections (i.e., about 50-80 islets) were evaluated per mouse in a blinded fashion by two independent observers who were blind to the experimental conditions. The degree of leukocytes infiltration in the pancreatic islets was used to score insulitis and quantified as follows: 0, no infiltration; 1, peripheral insulitis with or without minor infiltration (less than 20% of islet) 2, severe insulitis with clear islet infiltration (more than 20% of islet infiltrated) and histological signs of islet tissue damage.

### Statistical analysis

Results of the experimental studies are expressed as mean ± standard error of the mean (SEM). Statistical analyses were performed using GraphPad Prism (v.9.5.1). Student’s Ttest was used while comparing two groups. Browne-Forsythe and Welch one way ANOVA tests were used for analyzing data for more than two groups. Values of p < 0.05 were considered to be statistically significant.

## Results

### TIMP3 overexpression in myeloid cells improves islet morphology and insulin secretion in diabetic mice

**Figure 1:**
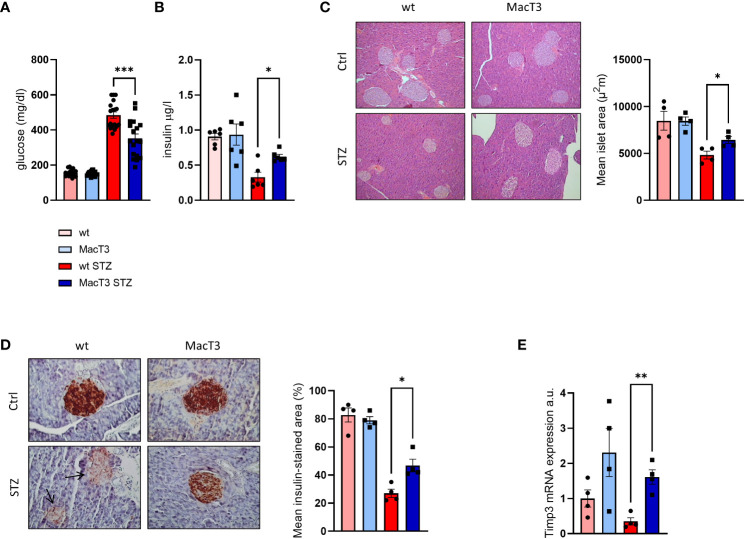
12 weeks after MLDS administration diabetic wt and MacT3 mice and age-matched non-diabetic littermates (Ctrl) were analyzed for **(A)** Blood glucose levels (n=18 per group) and **(B)** Serum insulin levels (n=6 per group), **(C)** Hematoxylin and eosin (H&E, scale bare = 150 μm) and **(D)** insulin-stained pancreatic sections (scale bar = 100 μm); n=4 per group. Islet area (H&E) and insulin-stained area (IHC) were determined analyzing three pancreatic sections 150 μm apart, and the results were expressed as mean islet area and % of islet area positive for insulin, respectively. All islets from the three pancreatic sections were measured, **(E)** pancreatic gene expression of TIMP3 (mRNA expression was determined by real-time PCR and normalized to β-actin mRNA) (n=4 per group) (*p < 0.05, **p ≤ 0.01, ***p ≤ 0.001; One-way ANOVA, data are means ± SEM). a.u., arbitrary unit.

8-week-old male wild type (wt) and MacT3 mice were treated with MLDS to induce diabetes. 12 weeks after MLDS blood glucose level was monitored and only diabetic mice were considered for further experiments. After 3 months of MLDS treatment, diabetic MacT3 mice showed a significantly reduction of glycaemia (**Figure 1A**), and increased serum insulin levels (**Figure 1B**), average islet size (**Figure 1C**), and insulin positive area (**Figure 1D**) compared to wt mice. mRNA expression of Timp3 was downregulated in pancreas of diabetic wt mice compared to non-diabetic, whereas MacT3 mice showed significantly higher levels of Timp3 in both conditions (**Figure 1E**) compared to wt mice, suggesting a relevant protective role of CD68 dependent TIMP3 in the preservation of islet morphology, insulin secretion and insulin storage.

### TIMP3 enhances islet vascularization in diabetic mice

**Figure 2:**
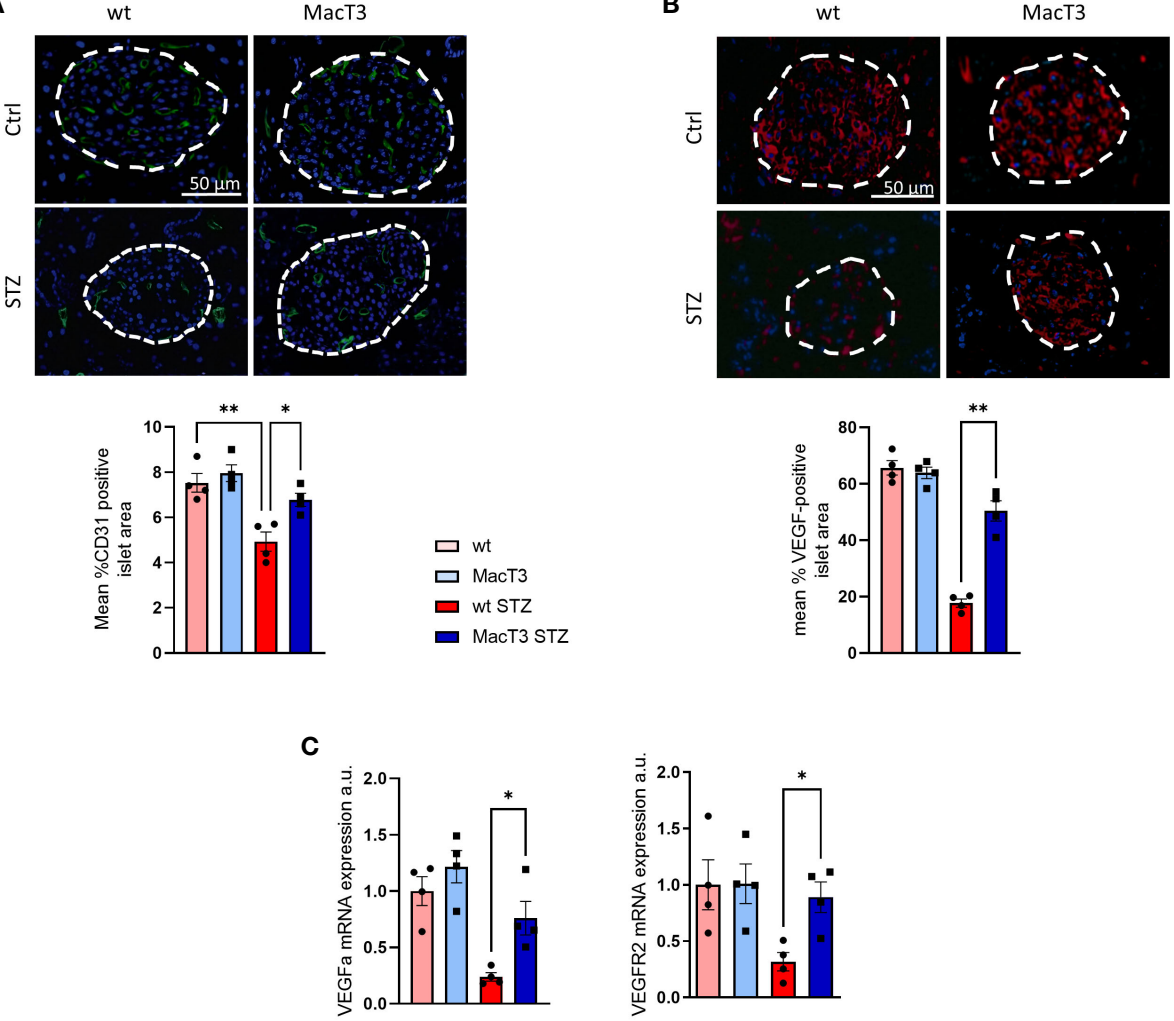
12 weeks after MLDS administration pancreas from diabetic wt and MacT3 mice and age-matched nondiabetic littermates (Ctrl) were analyzed for **(A)** CD31, **(B)** VEGF protein expression by immunofluorescence (scale bar = 50 μm, n = 4 per group). Fluorescence sections were examined at a final magnification of 400X. Islet capillary density and VEGF protein expression were measured as CD31 (green) and VEFG (red) positive area, respectively, determined by analyzing three pancreatic sections 150 μm apart, and the results were expressed as % of islet area positive for CD31 or VEGF. Islets are outlined with dashed white lines. **(C)** gene expression of VEGF-A and VGFR2 (mRNA expression was determined by real-time PCR and normalized to β-actin mRNA) (n=4 per group) (*p < 0.05, **p ≤ 0.01; One-way ANOVA, data are means ± SEM). a.u., arbitrary unit.

Impaired islets vascularization represents a relevant contributor for the STZ-induced progressive β-cell failure and mass depletion (12). Therefore, the expression pattern of CD31, a marker of endothelial cells and intraislet vasculature, and VEGF, a regulator of islet vascular development, was analyzed in pancreas. Results showed the reduction of CD31 and VEGF in wt diabetic mice compared to non-diabetic wt and MacT3 diabetic mice (**Figures 2A, B**). Consistently, mRNA expression of VEGF-A and its receptor VEGFR2 was significantly reduced in wt diabetic mice compared to MacT3 diabetic mice (**Figure 2C**), indicating a role for myeloid cell-derived TIMP3 in the regulation of islet vascularization in diabetic pancreas.

### TIMP3 is associated with improvement of antioxidant defense in diabetic mice

**Figure 3:**
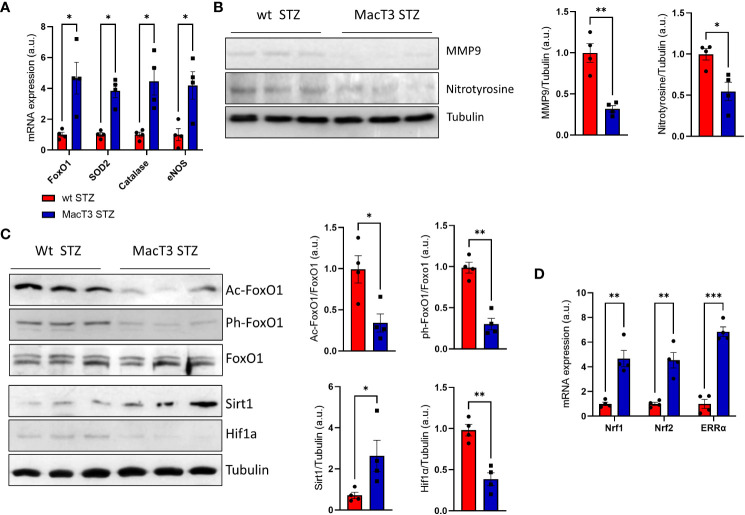
**(A)** gene expression of FoxO1, SOD2, Catalase and eNOS, **(B)** protein levels of MMP9, Nitrotyrosine and Tubulin, **(C)** Protein levels of FoxO1 acetylated, FoxO1 phosphorylated, total FoxO1, Sirt1, HIF-1a and Tubulin, **(D)** gene expression of Nrf1, Nrf2 and ERRa in pancreas of wt and MacT3 diabetic mice 12 weeks after MLDS administration (n= 4 per group). A representative image of 3 mice per group is shown. Relative protein levels as determined by densitometry (mRNA expression was determined by real-time PCR and normalized to β-actin mRNA) (*p < 0.05, **p ≤ 0.01, ***p ≤ 0.001; Student’s t test comparing diabetic mice, data are means ± SEM). a.u., arbitrary unit.

Pancreatic damage and dysfunction are strongly induced by oxidative stress (13). Thus, the effects of TIMP3 overexperession on the pancreatic redox status was analyzed. Pancreatic mRNA expression of antioxidant markers, such as FoxO1 and its targets SOD2, Catalase, and eNOS was significantly increased in diabetic MacT3 mice compared to diabetic wt mice (**Figure 3A**), whereas protein levels of the oxidative stress marker nitrotyrosine and of MMP9, which is upregulated by oxidative stress, were reduced (**Figure 3B**), suggesting an improvement of the antioxidant defenses associated to TIMP3 overexpression.

### Effects of TIMP3 on the expression of Sirt1 and its targets in diabetic pancreas

Transcriptional activity of FoxO1 is regulated by post-translational modification such as acetylation and phosphorylation and the acetylation dependent association between SirT1 and FoxO1 transcription factor leads to the activation of FoxO1 mechanisms involved on the oxidative stress resistance (14). Pancreas from MacT3 diabetic mice showed increased protein level of Sirt1 associated to reduced FoxO1 acetylation and phosphorylation compared to wt diabetic mice (**Figure 3C**). The increase of Sirt1 was also accompanied by reduced protein levels of the hypoxia-inducible factor 1α (HIF-1α) (**Figure 3C**), a well-known substrate for SIRT1 deacetylation (15) and by increased mRNA expression of nuclear respiratory factor 1 (Nrf-1), nuclear respiratory factor 2 (Nrf-2), estrogen-related receptor-α (ERR-α), all being involved in the control of mitochondrial biosynthesis and function (**Figure 3D**).

### TIMP3 ameliorates insulitis in STZ-induced diabetic mice

**Figure 4:**
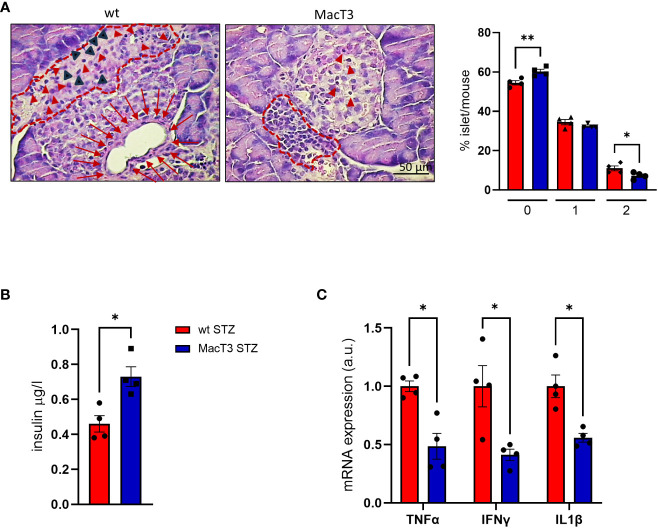
At 11th day after MLDS administration hematoxylin and eosin (H&E) pancreas sections from wt and MacT3 mice were analyzed for **(A)** insulitis severity by assessing the percentage of islets per mouse in each stage of insulitis using the following scores: 0, no infiltration; 1, peri-insulitis with or without minor infiltration (less than 20% of islet); 2, severe insulitis with clear islet infiltration. Representative photographs of mouse pancreas stained with hematoxylin and eosin show: in wt mice, partially destroyed islet (dotted line) with inflammatory infiltration. The normal endocrine tissue is replaced by amorphous material containing inflammatory (red arrowheads) and fibroblast-like cells (black arrowheads). Below, dilation of a pancreatic duct enveloped by a thin fibrotic reaction (red arrows) with compression of the islet parenchima. In MacT3 mice peripheral insulitis (dotted line) with minor infiltration (red arrowheads). **(B)** Serum insulin levels, **(C)** gene expression of TNFα, IFNγ and IL1β in pancreas. (n= 4 per group *p < 0.05, **p ≤ 0.01; Student’s t test comparing diabetic mice, data are means ± SEM). a.u., arbitrary unit.

Immune-cell infiltration into the islets of Langerhans (i.e., insulitis) is considered a hallmark of T1DM development and reflects the autoimmune nature of the disease. Since insulitis typically occurs in the early stages of T1DM, before the clinical symptoms (15), and 55% of MacT3 mice completely failed to develop T1DM, compared to 15% of wt (11), to assess whether the protection conferred by TIMP3 overexpression may involve the control of insulitis, the severity of insulitis was measured on the 11th day after MLSD treatment, when the infiltration of immune cells reaches its peak (16). Insulitis score, referred to the degree of pancreatic islet leukocytic infiltration, was significantly reduced in MacT3 mice compared to wt, whereas insulin level in serum was increased (**Figures 4A, B**). Moreover, the levels of pro-inflammatory cytokines, such as IL1β, TNFα and IFNγ in the pancreas, that mirror the gravity of injury in pancreatic islets (17), were significantly reduced in STZ-induced diabetic MacT3 mice compared with wt mice (**Figure 4C**).

## Discussion

We have previously demonstrated that MacT3 mice are protected against diabetes-induced kidney damage and albuminuria (11). During that study we also noticed that MacT3 mice show the 40% reduction of diabetes incidence after MLSD treatment (11). In this study we show that TIMP3 overexpression in myeloid lineage exerts a protective effect on MLSD induced pancreatic damage by ameliorating insulitis and pancreatic tissue inflammatory profile, thus improving islet morphology and insulin secretion. Furthermore, the protective effects of TIMP3 overexpression are also associated with increased markers of neovascularization, oxidative stress resistance and mitochondrial biogenesis after three months of diabetes, suggesting a role for TIMP3 in maintaining islet tissue homeostasis and/or recovering from STZ-induced injury.

The vascularization of pancreatic islets plays a vital role in their development and function and the deterioration of the pancreatic islet network of blood vessels throughout the course of diabetes progression has been documented (12). VEGF is important in the maintenance of islet microvasculature and β cells function, and inhibition of VEGF signaling results in rapid regression of the islet vasculature (18). Here we show that the islet microvasculature of MacT3 diabetic mice, was better conserved than in diabetic wt mice, as evidenced by CD31, VEGF and VEGFR2 expression. This finding indicates a role for myeloid-derived TIMP3 expression in regulating islet vascularization by supporting the VEGF system.

Pancreatic islets, characterized by a limited antioxidant capacity, are prone to oxidative stress that is closely related to pancreatic damage and T1DM progression (13).. Here we show an improvement of the antioxidant defenses associated to TIMP3 overexpression, as evidenced by increased expression of FoxO1 and its antioxidant targets. Consistently, phosphorylation of FoxO1 protein was reduced in diabetic MacT3 pancreas, suggesting increased nuclear localization and transcriptional activity.

It has been reported that FOXO1 deacetylation by SIRT1 favors its antioxidant transcriptional activity program in diabetic tissues (14). Besides decreasing oxidative stress, SIRT1 activity can also protect pancreatic islet function by improving mitochondrial biogenesis and regulating insulin secretion (19). Insulin secretion and nutrient sensing in β cells are thightly associated to appropriate function of mitochondria. Therefore, we speculate that in MacT3 mice the increase of pancreatic protein levels of SIRT1 may help preserve pancreatic endocrine function. Beside being associated with reduced acetylation of FoxO1. Sirt1 upregulation was associated with the restoration of the transcription factors Nrf1 and Nrf2 and the nuclear receptor ERR-α. Interestingly, Nrf1 is crucial for transcriptional control of genes implicated in oxidative phosphorylation and mitochondrial biogenesis (20), Nrf2 is involved in the regulation of a set of antioxidant and xenobiotic-metabolizing enzyme as well as glucose metabolism in diabetic β cells (21), and ERR-α is a transcriptional regulator of cellular energy metabolism that is suppressed in diabetic patients (22). Diabetic MacT3 mice also show reduced HIF-1α protein levels, a known substrate for SIRT1 deacetylation (15). This transcription factor is crucial in the cellular responses to altered oxygen level. Both disruption and overexpression of HIF-1α in β cells may result in detrimental effects on β cell function in mice, indicating that the strict control of HIF-1α may be crucial for proper β cell function maintenance (23). In addiction HIF-1α is known to be relevant in the energy metabolism regulation and can also be modulated by oxygen-independent stimuli, including metabolic factors such as insulin and glucose (24). Therefore, reduced HIF-1α levels in MacT3 diabetic mice versus wt diabetic mice may reflect the improvement of both tissue oxygen supply and metabolic factors.

The pro-inflammatory cytokines released by immune cells in and around islets of Langerhans during insulitis are involved in the onset of T1DM (25). Since TIMP3 shows anti-inflammatory properties in several contexts (7–11), we investigated the effect of TIMP3 overexpression on the early stages of T1DM development and found that, compared to wt diabetic mice, MacT3 diabetic mice showed reduced mRNA expression of TNFα, IFNγ and IL1β. This finding was paralleled by reduced insulitis and a marked increase in insulin production, indicating that myeloid TIMP3 could protect β cell function by improving the pancreatic inflammatory profile during insulitis. From a mechanistic standpoint, it is plausible to speculate that the upregulation of Timp3, through the inhibition of proteinase activity of different targets, could potentially mitigate tissue inflammation by influencing the migratory capacity of leukocytes. Future research will be required to localize specific proteolytic activity in pancreatic tissue sections to provide crucial additional information on the mechanism involved in the protection exerted by Timp3. The study’s primary limitation is the lack of analysis at an intermediate stage (e.g., one month after MLSD treatment), which would have provided insights into whether the improved islet morphology and vascularity observed at 3 months in diabetic MacT3 mice is solely due to reduced post-STZ insulitis or also influenced by the recovery ability from injury during the post-insulitis, prediabetic phase. Similarly, the lack of an intermediate analysis period hinders the establishment of a direct causal link between the overexpression of TIMP-3 and the enhanced preservation of VEGF-A. This result may arise from reduced destruction and preservation of the original beta cells, which are the primary source of VEGF-A in the islets. Other limitation of this study is that it was performed in whole pancreas instead of isolate beta islets. However, it is essential to acknowledge that STZ is known to be toxic to β-cells, provoking insulitis and causing damage to the pancreatic islets. Consequently, the observed alterations in gene expression patterns and protein levels eleven days after STZ treatment can be attributed to the effects of insulitis and islet damage. Therefore the observed changes would have been more pronounced and statistically significant had we undertaken an isolation of the pancreatic islets.

In conclusion, these results show that overexpression of myeloid TIMP3 protects from T1DM development by reducing pro-inflammatory cytokine production and STZ-induced insulitis, thus alleviating pancreatic damage. Improved islet (re)vascularization and oxidative stress homeostasis may also contribute to the protective effects on pancreatic β cells associated with TIMP3 overexpression. Further studies are still required to support these findings, elucidate additional molecular mechanisms, and understand their potential clinical relevance and translatability to human T1DM.

## Data availability statement

The original contributions presented in the study are included in the article/**Supplementary Material**. Further inquiries can be directed to the corresponding authors.

## Ethics statement

The animal study was approved by Ministry of Health, license no. 378/2016-PR and 36/2019-PR. The study was conducted in accordance with the local legislation and institutional requirements.

## Author contributions

VC: Data curation, Investigation, Methodology, Validation, Writing – original draft, Formal Analysis, Visualization. SM: Data curation, Formal Analysis, Investigation, Methodology, Validation, Funding acquisition, Writing – review & editing. CI: Formal Analysis, Investigation, Methodology, Validation. GP: Writing – review & editing, Supervision. MF: Supervision, Writing – review & editing, Conceptualization, Funding acquisition, Project administration. RM: Conceptualization, Project administration, Supervision, Data curation, Investigation, Methodology, Validation, Writing – original draft.
