# Supplementary material for: TIMP3 overexpression in myeloid lineage alleviates pancreatic damage and confers resistance to the development of type 1 diabetes in the MLDS -induced model
Source: Front Endocrinol (Lausanne). 2024 Jan 19;14:1297847. doi: 10.3389/fendo.2023.1297847 (PMC10835381; doi:10.3389/fendo.2023.1297847)
Supplement: Supplementary file 1 [file DataSheet_1.pdf]

TIMP3 overexpression in myeloid lineage alleviates pancreatic damage and confers resistance to the development of type 1 diabetes in the MLDS -induced model

Viviana Casagrande[1], Stefano Menini[2], Chiara Internò[1], Giuseppe Pugliese[2], Massimo Federici[1,3]*, Rossella Menghini[1]*

[1] Departments of Systems Medicine, University of Rome "Tor Vergata," Rome, Italy.

[2] Department of Clinical and Molecular Medicine, "Sapienza" University, Rome, Italy.

[3] Center for Atherosclerosis, Department of Medical Sciences Policlinico Tor Vergata University, Rome, Italy.

*Corresponding authors: E-mail: menghini@med.uniroma2.it (R.M.); federicm@uniroma2.it (M.F.)

Type 1 Diabetes Model and Experimental Protocols

Male C57Bl/6J (wt) (Charles River Laboratories) and MacT3 mice were maintained in our animal facility (12 hours light/dark cycle; 22◦C ± 1 ◦C, 50% ± 5% humidity) and fed ad libitum with standard laboratory chow (Mucedola s.r.l.). Genotyping of the animals was analyzed using polymerase chain reaction (PCR) on DNA isolated from the tail (9,10). Diabetes was induced by a multiple low-dose streptozotocin (STZ) (Merck, Darmstadt, Germany) injection. 8-week-old mice wt and MacT3 received either sodium citrate (control) or STZ (50 mg/kg, pH 4.5, dissolved in sodium citrate) through intraperitoneal injection for 5 consecutive days after 4 hours fast. Animals with a fed glucose level ≥250 mg/dL, measured by using a glucometer (OneTouch Ultra, LifeScan, Malverne, USA), have been considered diabetic. Mice were sacrificed 12 weeks after the final injection of STZ (11). The sacrifice of the animals, carried out at the end of each experiment, was performed by cervical dislocation; the biochemical and morphological analyzes were carried out on blood and organs taken at the time of the sacrifice. Measurements of plasma insulin was obtained using an ultrasensitive mouse insulin ELISA kit (Mercodia, Uppsala, Sweden) according to the manufacture's protocol.

Histopathological, Immunohistochemical and Immunofluorescence analysis

Embedded pancreas were sectioned at 4 µm thickness. Haematoxylin and eosin-stained (H&E) pancreas sections from mice after 11 days from the MLSD treatment were evaluated for the insulitis score and pancreas section from mice after 3 months from the MLSD (diabetic mice) or vehicle (control mice) treatment were used to measure the mean islet area and insulin-positive islet area by IHC. For insulin staining, the primary antibody used was an anti-Insulin guinea pig polyclonal (Abcam, Cambridge, UK) diluted 1:200, followed by a secondary byotinilated goat anti-guinea pig (Abcam) diluted 1:1000. Sections were examined using a Nikon Eclipse E600 light microscope (Nikon, Inc., Tokyo, Japan). For CD31 and VEGF staining, the primary antibodies used were a rabbit monoclonal to PECAM-1 (Abcam) diluted 1:2000 and a mouse monoclonal to VEGF (Invitrogen Corp, Eugene, OR) diluted 1:100, followed by the fluorescent-labeled antibodies DyLight®488 anti-rabbit IgG (Vector Labs, DI-1488) and DyLight™ 594 Anti-Mouse IgG (Vector Labs, DI-2594), respectively, at the final concentration of 10 µg/ml. Images were acquired using a 25x/N.A. 0.95 on a Zeiss Axiovert 200 M fluorescence microscope equipped with an Axiocam 503 color camera, controlled with ZEN (blue edition) software (Zeiss, Milan, Italy).

Morphometric Analysis of Pancreatic Islets

Section of pancreas stained with H&E and immunostained for insulin, CD31, and VEGF were examined at a final magnification of 400X. The mean islet area and the percentage if islet area positive for insulin, CD31 (i.e., islet capillary density), and were determined analyzing three pancreatic sections 150 µm apart and calculated by means of the image analysis system Image Pro Premier 9.2. The results were expressed as mean islet area and % of islet area positive for insulin, CD31, and VEGF, respectively.

Immunoblotting

Pancreas were homogenized in ice cold buffer containing 20 mM Tris (pH 7.6), 137 mM NaCl, 1 mM MgCl2, 1 mM CaCl2, 1% Triton X-100, 10% Glycerol, 2 mM EDTA plus protease inhibitors, for 20 minutes at 4°C, and the remaining debris was cleared by subsequent 20 minutes centrifugation at 12500 rpm, 4°C. The protein concentrations were evaluated by Bradford (BioRad, Hercules, California, USA); 40 µg of total protein per lane was diluted in standard SDS sample buffer and subjected to electrophoresis on SDS polyacrylamide gels. The following antibodies were used: MMP9, FoxO1, HIF-1α (Santa Cruz Biotechnology, Dallas, Texas; USA); Nitrotyrosine, Phospho-FoxO1 (Ser256), Acetylated-FoxO1 (Cell Signaling Technology, Danvers, Massachusetts, USA); α-Tubulin (Thermo Fisher Scientific, Carlsbad, California, USA); SIRT1 (Abcam). Quantity One/ImageJ sofware was used for densitometric scanning.

RNA Isolation and Gene Expression Analysis

Total RNA was isolated from pancreas with Trizol reagent (Invitrogen) and quantified by Nanodrop (Thermo Fisher Scientific). 2 μg of RNA was reverse transcripted into cDNA using the High Capacity cDNA Archive Kit). Quantitative real-time PCR was performed, using an ABI PRISM 7700 System and TaqMan reagents (Applied Biosystems), and $2^{-\Delta\Delta Ct}$ was calculated to have the relative gene copy number. Of each reaction a triplicate was made by appling the same standard conditions: 1 cycle at 50°C for 2 minutes, 1 cycle at 95°C for 10 minutes, and 40 cycles each at 95°C for 15 seconds and 60°C for 1 minute. The cycle threshold value was normalized in mouse by 18S. Primer codes (Applied Biosystems): mTIMP3; mVEGFR2; mVEGFa; mFOXO1; mSOD2; mCAT; m-eNOS; mTNFα; mIFNγ; mIL1β; mNRF1; mNRF2; mERRα.
